# Feature tracking cardiac magnetic resonance in systemic light chain amyloidosis

**DOI:** 10.1186/1532-429X-15-S1-P97

**Published:** 2013-01-30

**Authors:** Rebekka Kammerer, Kristin Breuninger, Philipp Matheis, Yannick Sander, Katrin A Scherer, Lukas Rust, Christian Galuschky, Evangelos Giannitsis, Arnt V Kristen, Grigorios Korosoglou, Sebastian Buss

**Affiliations:** 1 University of Heidelberg, Heidelberg, Germany; 2 TomTec Imaging Systems, Munich, Germany

## Background

Systemic light chain amyloidosis (AL) is associated with a high cardiovascular morbidity and mortality. Cardiovascular involvement and determination of prognosis is underestimated by standard imaging parameters. Recently, cardiac deformation analysis of global circumferential and longitudinal strain has been shown to have great clinical impact on the assessment of prognosis and survival in this rare disease. For quantification of cardiac deformation analysis we applied a novel non-invasive post-processing feature tracking algorithm (FTI) on pre-acquired regular CMR SSFP images in healthy volunteers and in patients with AL and sought to investigate wall motion differences between both groups.

## Methods

65 patients (mean age 58 ± 11 years; 41 male, 24 female patients) with biopsy proven systemic AL were scanned on a clinical 1.5 T CMR scanner (Philips Achieva). Short axis slices covering entirely both ventricles as well as 2-, 3- and 4-chamber were acquired using standard SSFP-sequences before initiation of specific pharmaceutical AL therapies. The control group consisted of 50 healthy subjects (mean age 58 ± 5 years; 23 male, 27 female). Besides the standard CMR parameters for volumes, ejection fraction (EF) and myocardial mass and wall thickness we measured global circumferential and longitudinal strain on SSFP images by the application of the post-processing feature tracking algorithm.

## Results

Global circumferential strain and global longitudinal strain correlated well with left ventricular ejection fraction (r^2=0.64, p<0.05; r^2=0.47, p<0.05). In patients with AL global longitudinal strain was significantly reduced compared to healthy subjects (-16.9±5.1% vs -23±3.3%, p<0.05), whereas global circumferential strain was not (-25.1±7.0% vs -27.1±5.0%, n.s.). In the subgroup analysis of AL patients without cardiac involvement (mean wall thickness ≤ 12mm) global longitudinal strain showed significantly reduced values in comparison to healthy control subjects (-20.3±4.7% vs -23±3.3%, p<0.05), whereas global circumferential strain did not show a significant difference. Patients with an ejection fraction ≥50% already had reduced global longitudinal strains (-18.5±4.5% vs -23±3.3%, p<0.05), again global circumferential strain did not show a significant difference.

## Conclusions

FTI strain analysis derived from regular SSFP sequences offers the possibility for a retrospective or prospective fast quantitative wall motion assessment of myocardial deformation patterns without specific and time-consuming strain imaging techniques. FTI allows distinguishing between healthy subjects and subjects with AL. Further investigations are necessary to analyze the impact of this new method on clinical outcome in AL patients.

## Funding

none

